# Molecular Nevogenesis

**DOI:** 10.1155/2011/463184

**Published:** 2011-04-06

**Authors:** Andrew L. Ross, Margaret I. Sanchez, James M. Grichnik

**Affiliations:** Department of Dermatology and Cutaneous Surgery, University of Miami Miller School of Medicine, Miami, FL 33136, USA

## Abstract

Despite recent advances, the biology underlying nevogenesis remains unclear. Activating mutations in NRAS, HRAS, BRAF, and GNAQ have been identified in benign nevi. Their presence roughly correlates with congenital, Spitz, acquired, and blue nevi, respectively. These mutations are likely to play a critical role in driving nevogenesis. While each mutation is able to activate the MAP kinase pathway, they also interact with a host of different proteins in other pathways. The different melanocytic developmental pathways activated by each mutation cause the cells to migrate, proliferate, and differentiate to different extents within the skin. This causes each mutation to give rise to a characteristic growth pattern. The exact location and differentiation state of the cell of origin for benign moles remains to be discovered. Further research is necessary to fully understand nevus development given that most of the same developmental pathways are also present in melanoma.

## 1. Introduction

Nevogenesis is a multifactorial process that involves a complex interplay of genetic and environmental factors. Although we are only just beginning to understand this process, it is already clear that certain molecular pathways within nevocytes need to be activated in order for nevogenesis to occur. This paper will focus on the relevant identified pathways that promote the development of the different nevus phenotypes.

## 2. Nevus Life Cycle

Benign melanocytic lesions follow an archetypal life cycle that consists of four stages: initiation, promotion, senescence, and involution. Initiation occurs when a nevus progenitor cell acquires a mutation that will permit future growth. Promotion occurs when the mutated cell is activated and proliferation begins. This proliferation is likely instigated by a change in local environmental factors that promotes melanocytic growth and then sustained by the previously acquired mutation. After a period of growth, nevi stop proliferating through the activation of senescence pathways. This allows them to remain stable for extended periods of time before undergoing involution. 

## 3. Models for Nevogenesis

Current models of nevogenesis propose that melanocytic neoplasms arise from a single cell of origin [1, 2]. However, the differentiation state of this cell has not been clearly established. It is also uncertain if the progenitor cell is located in the dermis, epidermis, or both.

One possibility is that an immature melanocytic stem cell serves as the nevus progenitor cell. Although this progenitor cell most likely resides in the dermis, its presence in the epidermis can not be excluded. In this model, the immature cell remains in a quiescent state in the skin and acquires mutations secondary to UV light exposure or other mutagenic processes. When environmental signals activate this cell to produce melanocytes, an abnormal proliferation occurs due to the genetic alterations. The specific underlying mutation and local environmental conditions alter the daughter cells' normal melanocytic differentiation and migratory pathways in a characteristic manner. This causes the nevus to assume a discrete phenotypic pattern.

One of the advantages of this immature progenitor cell model is that the mutated cells can remain quiescent until activated. This readily explains the association of childhood sun exposure with the development of nevi and melanoma later in life. It would also explain the phenomenon of eruptive nevi simultaneously growing in response to a cytokine or an immunoregulatory medication. Consequently, this model seems to fit best with clinical findings.

It has also been suggested that a differentiated melanocyte serves as the cell of origin for melanocytic neoplasms. In this model, the nevogenic mutation occurs in a differentiated melanocyte. The mutation causes the cell to regain proliferative capacity. However, this genetic event would also have to promote dedifferentiation and the development of invasive properties in order to allow the cell to migrate to greater depths in the dermis. In this model, nevus growth would occur immediately after the initial mutagenic event in either a fast or slow manner. This is somewhat more difficult to reconcile with the clinical behavior of nevi.

As previously mentioned, both of these models are based on nevi arising from a single cell of origin. The concept of monoclonal origination is supported by the fact that NRAS and BRAF mutations are almost always mutually exclusive. However, recent studies on BRAF (reviewed below) have documented mutation heterogeneity within nevi. There exist at least two explanations for this phenomenon. First, an unidentified primary mutation that impacts DNA synthesis and repair machinery could make melanocytes more susceptible to developing BRAF mutations. Alternatively, local environmental conditions could lead to the recruitment of cells with different mutations to the lesion (hamartoma). Although a hamartoma can not be excluded, it seems likely that there are unidentified processes occurring within these cells that drive the heterogeneity of BRAF mutations.

## 4. NRAS and Congenital Nevi

**Figure 1:**
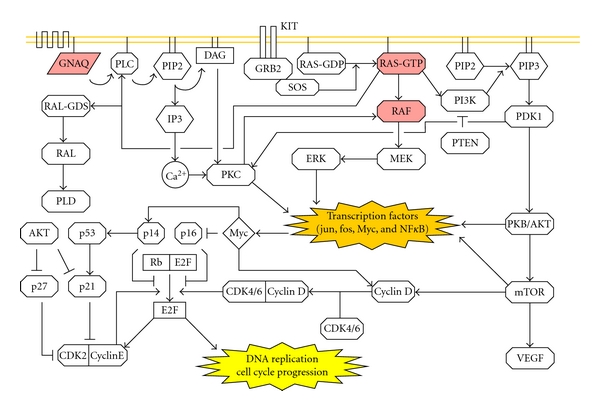
Relative position of RAS, RAF, and GNAQ in intracellular signaling. Mutations in NRAS, HRAS<BRAF, and GNAQ lead to alterations in transcription factor expression (MIT-F and Brn2 not shown). This in turn drives a number of processes like cell cycle progression. The contribution of the transcription factor Myc to cell cycle progression and its relationship to tumor suppressors are shown downstream of the aforementioned mutations.

NRAS is one of the three major isoforms of the RAS family of GTPase proteins that are involved in cell growth, differentiation, and survival. NRAS activates four major signaling pathways: (1) RAF-MEK-ERK, (2) RalGDS, (3) PI3K-AKT/PDK1, and (4) PLC/PKC (see Figure 1). Activation of these pathways results in a variety of different outcomes that include cell cycle progression, upregulated transcription, upregulated translation, nuclear transport, and calcium signaling. All reported NRAS gene mutations occur in exon 2 and exon 3 (known before as exon 1 and exon 2). Approximately 65% of the mutations occur at codon 61 in exon 3, where the most frequent amino acids substitutions are Q61K and Q61R [14]. The replacement of the glutamine residue (Q61) with lysine (K) or arginine (R) results in an aberrant protein that is unable to cleave GTP, and thus the protein remains constitutively active. Although NRAS mutations have been reported in other melanocytic nevi, the mutation seems to be most closely associated with congenital melanocytic neoplasms (see Table 1) [11, 15–17].

In 1994, Carr and Mackie provided the first report documenting the presence of an activating NRAS mutation in 28% (12 of 43) of congenital melanocytic nevi (CMN) [3]. This finding was confirmed by subsequent studies [4, 5]. Following this discovery, BRAF mutations were also reported in CMN with incidences ranging between 39 and 86% [6–8]. However, it is important to note that all of the aforementioned studies relied solely on histological appearance to classify the lesions as CMN. As such, there was no evidence that these lesions had been present at birth. Thus, many of the specimens likely represented acquired nevi with histological features of congenital nevi. Bauer et al. demonstrated that using nevi with histological features of CMN but no documentation of presence at birth introduces a selection bias that artificially increases the incidence of BRAF mutations in CMN [11]. Reaves et al. addressed this issue by selecting CMN specimens whose presence were documented in medical records at birth. None of the 36 medium and large CMN in this series possessed BRAF mutations [9]. 

The following year Ichii-Nakato et al. found that 79% (33 of 42) of small CMN and 30% (6 of 20) of medium CMN present at birth by records or parents testimony were BRAF positive [10]. This study demonstrated a statistically significant difference in the BRAF mutation rate between small- and medium- sized CMN. The recent studies that document CMN presence at birth have reported NRAS mutations in 81% (26 of 32) [11] and 70% (19 of 27) [11, 13] of the CMN. BRAF mutations were found in 0% (0 of 32) [11] and 22% (6 of 27) [13] of the nevi. One report noted that 76% (26 of 34) [12] of CMN had BRAF mutations; however, the study contained a disproportionately high number of small CMN.

Overall, NRAS mutations were found to exist in 55% (77 of 141) of the CMN specimens specifically assayed for genetic NRAS abnormalities, making it the most common mutation in CMN. BRAF mutations are also common, occurring in 39.3% (90 of 229) of the CMN specifically assayed for genetic BRAF abnormalities. NRAS mutations clustered in medium and large CMN (66%; 62 of 94 specimens studied) while BRAF mutations clustered more frequently in the small CMN (79%; 53 of 67 specimens documented as <1.5 cm). The latter association may be due to the inclusion of acquired nevi with histological features of congenital nevi and/or a difference in the growth potential between the two genes.

The initiating event that causes the NRAS mutation is not yet known. Nevertheless, it is clear that UV radiation does not play a role in initiation given that the mutation occurs *in utero*. It is also uncertain which type of cell undergoes initiation, though it likely occurs in a neural crest stem cell. The resultant disregulation of the neural crest stem cell during development then leads to a massive deposition of nevocytes along its native migration pathways to the epidermis. The presence of NRAS mutant cells in the dermis also appears to alter local environmental conditions as evidenced by the presence of longer and darker hair than the surrounding skin. BRAF mutations may also give rise to congenital nevi, though these lesions tend to be smaller. This may be due to the way the mutations function. NRAS mutations favor upregulation of the CRAF isoform of RAF over the BRAF isoform [30]. Although CRAF and BRAF exhibit a significant amount of functional redundancy, they have been shown to possess some unique functions [31]. One important example of this is CRAF's ability to inhibit apoptosis [32]. Theoretically, inhibition of apoptosis could allow for a greater expansion of melanocytes and thus account for the association of NRAS mutations with larger CMN. 

In summary, activating NRAS mutations appear to be most closely associated with the development of congenital melanocytic nevi. This association is accentuated in the larger lesions. BRAF mutations may also be associated with the development of CMN. Lastly, it is likely that there remain other unidentified mutations that contribute to the development of CMN.

## 5. HRAS and Spitz Nevi

HRAS is another member of the RAS protein family that is involved in signal transduction from the cell surface to the nucleus. Like NRAS, HRAS is able to activate all of the same aforementioned pathways (see Figure 1). HRAS mutations in exon 2 and exon 3 have previously been reported in melanocytic lesions [17, 19]. The most frequently reported mutation involves the replacement of a glutamine at residue 61 (Q61) of exon 3 with a lysine(K) residue [17]. This leads to the production of an aberrant protein that can not be deactivated and thus stimulates cell growth and differentiation. 

Genetic anomalies in HRAS occur almost exclusively in Spitz nevi (see Table 2). To date, no studies have been able to demonstrate the presence of genetic HRAS abnormalities in common acquired nevi, congenital nevi, or dysplastic nevi [38, 39]. Bastian et al. first noted HRAS copy number amplifications in 24% (4 of 17) of unequivocally diagnosed Spitz nevi in 1999 using comparative genomic hybridization analysis [18]. A second larger study by Bastian et al. not only demonstrated the presence of increased HRAS copy numbers in 12% (12 of 102) of Spitz nevi but also established the presence of HRAS mutations in 67% (8 of 12) of the Spitz nevi with increased copy numbers [19]. 

In 2003, while trying to ascertain the specificity of the archetypal V600E BRAF mutation as a marker for melanoma, Yazdi et al. demonstrated the absence of BRAF mutations in the 69 Spitz nevi studied [7]. This finding was confirmed in multiple studies over the next two years [17, 20–24]. In the 220 Spitz nevi analyzed in these experiments, not one harbored a BRAF mutation (see Table 2). All but one of the studies [22] utilized microdissection techniques of melanocytes from histological samples to decrease the rate of false negative results. However, only one study went as far to differentiate and include atypical Spitz nevi in the analysis [17].

The following year two papers challenged the notion that Spitz nevi lack BRAF mutations, showing that 21% (10 of 48) [25] and 100% (8 of 8) [26] of the specimens studied were indeed V600E BRAF positive. However, as previously pointed out [27, 29], one study exclusively examined Reed nevi while the other included more atypical inflammatory Spitz nevi that likely represented dysplastic nevi. Consequently, it is possible that variations in histological selection criteria created the variation in V600E BRAF positivity. This underscores the notion that current histological evaluations may not be the optimal method to classify nevi. Despite the flaws with these studies, it is not easy to dismiss their findings given that Da Forno et al. and Emley et al. were able to demonstrate the presence of a V600E BRAF mutation in a total of 7% (3 of 42) of unequivocal Spitz nevi studied [28, 29]. It should also be noted that 4% (4 of 98) of Spitz nevi studied by various groups also harbored a mutation in NRAS [17, 22, 25, 27–29]. 

In the series reviewed, HRAS mutations and gene amplification were noted to be present in 0–24% of Spitz nevi (see Table 2). However, it is likely that the reported incidence of HRAS mutations has been artificially deflated by the inclusion of certain nevi with histological classifications that are similar but likely unrelated to Spitz nevi in the literature. For example, when all of the specimens studied in Table 2 are considered, 49% (24 of 49) of genetic abnormalities identified occurred in HRAS. However, when spindle cell nevi of Reed are excluded from the specimens considered in Table 2, HRAS-associated anomalies are found to comprise 67% (24 of 36) of the genetic abnormalities identified. 

The initiating events that lead to the development of HRAS mutations are unknown. It is also unknown if the progenitor cell that sustains the mutation resides in the dermis or the epidermis, though it most likely resides in the dermis. Despite our lack of knowledge regarding HRAS initiation, it is clear that this mutation is associated with a specific nevus phenotype. This is likely related to the fact that HRAS has a significantly higher affinity for the PI3K-PKB/AKT pathway when compared to other RAS isoforms [41]. It would follow that preferential PI3K-PKB/AKT activation through HRAS drives the symmetrical overgrowth of cells with an epitheliod morphology without marked activation of the melanizing pathways (since the majority Spitz nevi are largely amelanotic). 

In summary, approximately 13% (24 out of 180) of unequivocal Spitz nevi studied have been shown to possess genetic HRAS anomalies. Although the majority of genetic abnormalities found in Spitz nevi involve HRAS, it is still only present in small fraction of the lesions studied. Consequently, it appears that HRAS is not the lone driving force responsible for the gross and microscopic characteristics of Spitz nevi. It is possible that unidentified mutations in other proteins such as those present in the PI3K-PKB/AKT pathway are responsible for the majority of Spitz nevi. 

## 6. BRAF and Melanocytic Neoplasms

BRAF is a serine-threonine kinase that is activated by the RAS family of proteins. Once activated, it triggers the BRAF-MAP-ERK signaling cascade that results in the upregulation of proteins that lead to cell cycle progression, transcription, and differentiation. This in turn promotes cell growth. Although over thirty BRAF mutations are known to exist, V600E is by far the most common variant found in melanocytic neoplasms [42, 43]. In the BRAF V600E mutation, a thymine at nucleotide 1799 on exon 15 is converted into adenine, causing glutamic acid to replace valine at residue 600. While BRAF V600E mutations have been described in congenital nevi [13], Spitz nevi [17], and blue nevi [22], they have been reported to occur with greatest frequency in acquired nevi (see Table 3) [12]. 

BRAF mutations were first described in 81% (57 of 70) of acquired nevi studied by Pollock et al. in 2003 [6]. Since this initial report, hundreds of acquired nevi have been analyzed for the presence of this mutation. Overall, almost 79% (376 of 479) of the acquired nevi studied were found to possess a mutation in BRAF. Unfortunately, not all of the papers went on to subdivide the acquired nevi into their histological subtypes (junctional, dermal, compound, etc.). When the available data is compiled from the studies in Table 3, 53% (19 of 36) of compound nevi [6, 37], 66% (2 of 3) of junctional nevi [6, 37], 65% (17 of 26) of dysplastic/atypical nevi [6, 35], and 83% (40 of 48) of intradermal nevi [6, 37] were found to possess BRAF mutations. No valid conclusions can be made with regards to the relative prevalence of BRAF mutations presented in each subtype due to selection bias induced by the exclusion of studies that did not publish histological subtype. Of note, NRAS mutations were described in 4.6% (5 of 108) of acquired nevi specifically assayed for the mutation [22, 33, 34, 37]. Less than 1% (1 of 108) of the nevi studied in these series were found to possess both BRAF and NRAS mutations [22, 33, 34, 37]. This is consistent with the notion that BRAF and NRAS mutations are almost always mutually exclusive.

The initiating events that lead to the acquisition of the BRAF V600E mutation are not known. Despite this, there is compelling evidence that the incidence of BRAF V600E mutations is not associated with the cumulative dose of UV exposure. Supporting evidence includes (1) studies that have shown that BRAF mutations occur more frequently in areas of intermittent sun exposure than areas of chronic sun exposure [34, 44]; (2) the fact that the V600E mutant lacks a classic UV signature [42]; and (3) reports that have characterized V600E mutations in CMN that developed *in utero * [12]. Nevertheless, these observations do not preclude UV exposure from contributing to the development of the V600E mutation. This is supported by the fact that BRAF mutations have been associated with UV exposure at a young age [45]. 

Thomas et al. have proposed an alternative mechanism for UV-induced BRAF mutations. It involves UVA radiation generating reactive oxygen species (ROS) that lead to the formation of cyclobutane pyrimidine dimers adjacent to nucleotide 1799. When replicative polymerase delta gets stuck at the adjacent cyclobutane pyrimidine dimer, error-prone polymerase eta assumes polymerase delta's replicative duties. This in turn would result in the commonly found T1799A substitution [45]. If this hypothesis is true, then the generation of ROS through any mechanism should produce BRAF V600E mutations. It is thus interesting to note an association between certain melanocortin-1 receptor (MC1R) polymorphisms and the incidence of BRAF V600E mutations [46]. This is because certain MC1R polymorphisms lead to increased pheomelanin production [47, 48], and pheomelanin is known to trigger ROS production [49]. 

It would be convenient to ascribe the molecular nevogenesis of acquired nevi to a single clonal population that developed from an oncogenic BRAF V600E-mutation in a single precursor cell. Unfortunately, current evidence argues against this. It has been shown that the percentage of total mutant alleles in BRAF V600E positive nevi varies from 5 and 40% [37]. This is much less than the 50% value that would be seen in a population of cells derived from a single heterozygous clone. Additionally, Lin et al. demonstrated that BRAF V600E mutations within single nevi were often heterozygous for a nearby single nucleotide polymorphism [50]. This suggests that the mutation must have occurred at least twice, once on each sister chromosome. Taken together, the data indicates that BRAF mutations do not represent an initiating event of nevogenesis. Instead, it is more likely that the mutation develops as part of a secondary process and may drive the promotion stage of nevogenesis. The presence of polyclonal BRAF V600E mutations also favors the supposition that an error-prone polymerase may be responsible for inserting an adenosine at nucelotide 1799 when it is invoked by oxidative stress. It is also possible that a different initiating mutation will be identified that creates a pro-BRAF V600E mutagenic environment in the acquired nevi. This would allow for a single clonal population especially prone to V600E mutations to be responsible for nevogenesis.

In summary, approximately 79% (376 of 479) of acquired nevi harbor BRAF mutations. The polyclonal nature of these BRAF mutations suggests that they represent a secondary event driving the promotion phase of nevogenesis. As such, the underlying initiating events that drive nevogenesis in acquired nevi remain to be identified.

## 7. GNAQ and Blue Nevi

GNAQ encodes an alpha subunit of the heterotrimeric G-protein responsible for transducing signals between G-protein couples receptors (GPCRS) and their downstream effectors. These effectors include but are not limited to the PLC/PKC and RAF-MEK-ERK pathways that also sit downstream of RAS [51]. GNAQ mutations almost exclusively involve the replacement of a glutamine residue at codon 209 of exon 5 by either leucine (51%) or proline (47%) residues [40, 52–54]. The affected residue is present in the ras-like domain of GNAQ and corresponds to the Q61 mutation found in NRAS and HRAS [40]. 

Few investigators have studied the genetic profiles of blue nevi. Combined data from these studies indicates that GNAQ is by far the most common genetic anomaly (82.8%); BRAF V600E mutations are rare (6.7%); NRAS mutations have not been have not yet been identified (see Table 4) [7, 22, 40]. Van Raamsdonk et al. have also reported one GNAQ mutation in seventeen nevi of Ota studied [40]. No studies have identified the presence of GNAQ mutations in other benign melanocytic tumors [39].

The initiating events that lead to the acquisition of GNAQ mutations are unknown. Although this mutation does not bear a classic UV signature, oxidative stress, possibly from UVA exposure, could play a role. The GNAQ mutation most likely occurs in a dermal melanocytic precursor as no epidermal component is present in the majority of lesions. This is consistent with what is known to occur in mice where the GNAQ mutation has been shown to lead to the accumulation of dermal melanin [51].

## 8. Conclusion

All of the previously discussed activating mutations occur in proteins that are components of complex cellular transduction pathways that exhibit a fair amount of overlap and redundancy (see Figure 1). As a result, NRAS, HRAS, BRAF, and GNAQ are all able to alter expression patterns of multiple transcription factors like Jun, Fos, Myc, NF*κ*B, Brn2, and MITF. These transcription factors in turn drive proliferation, differentiation, and senescence [55–57]. Despite the fact that NRAS, HRAS, BRAF, and GNAQ are involved in convergent pathways, mutations in these genes result in different cellular effects. This can be attributed to the various positions of each gene product in the pathways and different specificities for downstream effector molecules [41, 58]. This in turn results in each specific mutation generating its own characteristic growth pattern. The growth patterns may be further influenced by the location and the developmental stage of the cell of origin. 

The role of each of these mutations in nevus initiation and promotion has yet to be fully elucidated. It is likely that the acquisition of NRAS, HRAS, and GNAQ mutations represents the initiating event that primes a nevus progenitor cell to respond abnormally to normal melanocyte recruitment signals. Mutations in BRAF are more likely to represent secondary events in nevogenesis given their polyclonal nature. As such, BRAF mutations probably play a major role in acquired nevus growth promotion. 

NRAS, HRAS, and BRAF are all components of the mitogenic RAS-RAF-MEK-ERK pathway known to promote cellular proliferation [55]. GNAQ is also able to upregulate the RAF-MEK-ERK pathway via PKC [52]. Consequently, mutations in all of the respective gene products have the potential to promote melanocytic proliferation. However, it is also possible these mutations function to sustain growth promotion once another, earlier event, such as a change in extracellular signaling, induces the recruitment of the mutated melanocyte. 

All of these mutations have also been described in melanoma. The discriminating feature that distinguishes these benign nevi from melanoma is the induction of senescence pathways that signal growth arrests. This terminates the promotion phase and prevents indefinite proliferation. 

In conclusion, NRAS, HRAS, BRAF, and GNAQ have been identified in benign nevi, and their presence roughly correlates with congenital, Spitz, acquired, and blue nevi, respectively, (Table 5). In the future, dermoscopy and other noninvasive invivo imaging technologies may allow us to better predict which lesions harbor a specific mutation. There is still much to be learned about how these mutations occur, as well as the developmental stage and location of the progenitor cell. Since these mutations can also be found in melanoma, a further understanding of nevogenesis will have a direct impact on melanoma research efforts.

## Figures and Tables

**Table 1:** Congenital melanocytic nevi.

Study	Size studied	NRAS	BRAF	Specimen selection
Carr and Mackie 1994 [3]	Total^a^	12 of 43	n/a	Present before age 2 per parent testimony

Jafari et al. 1995 [4]	Total^a^	1 of 1	n/a	Histology alone

Papp et al. 1999 [5]	Total	10 of 18		Histology alone
	Small	2 of 3	n/a	
	Medium	8 of 15		

Pollock et al. 2003 [6]	Total^a^	n/a	6 of 7	Histology alone

Yazdi et al. 2003 [7]	Total^a^	n/a	6 of 13	Histology alone

Papp et al. 2005 [8]	Total^a^	n/a	7 of 18	Histology alone

Da Raeve et al. 2006 [9]	Total	n/a	0 of 36	Documented in medical records at birth
	Medium		0 of 10	
	Large		0 of 26	

Ichii-Nakata et al. 2006 [10]	Total	9 of 20	39 of 62	Documented in medical records at birth per parent testimony
	Small	n/a	33 of 42	
	Medium	9 of 20	6 of 20	

Bauer et al. 2007 [11]	Total^b^	26 of 32	0 of 32	Documented in medical records at birth

Bauer et al. 2007 [11]	Total^c,d^	7 of 28	20 of 28	Histology alone

Wu et al. 2007 [12]	Total	n/a	26 of 34	Documented in medical records at birth
	Small		20 of 25	
	Large		6 of 9	

	Total	19 of 27	6 of 27	Documented in medical records at birth
Dessars et al. 2009 [13]	Medium	1 of 3	1 of 3	
	Large	18 of 24	5 of 24^e^	

Totals	Total	77 of 141 (54.6%)	90 of 229 (39.3%)	
	Small	n/a	53 of 67 (79.1%)	
	Medium and Large	62 of 94 (65.9%)	18 of 124 (14.5%)	

(a) Size of specimens not specified; (b) all specimens either medium or large CMN; (c) all specimens small CMN; (d) not included in totals because purposely selected for acquired nevi with congenital patterns; and (e) two of the five represent chromosomal translocation involving BRAF.

**Table 2:** Spitz nevi.

Study	HRAS	NRAS	BRAF	Histological Subtypes Included
Bastian et al. 1999 [18]	4^a^ of 17	n/a	n/a	Typical Spitz nevi
Bastian et al. 2000 [19]	12^b^ of 102	n/a	n/a	Typical Spitz nevi
Yazdi et al. 2003 [7]^c^	n/a	n/a	0 of 69	Typical Spitz nevi
Palmedo et al. 2004 [20]^c^	n/a	n/a	0 of 21	Typical Spitz nevi
Mihic-Probst et al. 2004 [21]^c^	n/a	n/a	0 of 20	Typical Spitz nevi
Saldanha et al. 2004 [22]^d^	n/a	1 of 16	0 of 26	Typical Spitz nevi
Gill et al. 2004 [23]^e^	n/a	n/a	0 of 30	Typical Spitz nevi
Turner et al. 2005 [24]^d^	n/a	n/a	0 of 24	Typical Spitz nevi
Van Dijk et al. 2005 [17]^e^	6^f^ of 30	0 of 30	0 of 30	Typical and Atypical Spitz nevi
Fullen et al. 2006 [25]	n/a	1 of 48	10^g^ of 48	Spitz nevi and nevi of Reed
La Porta et al. 2006 [26]	n/a	n/a	8 of 8	Reed nevi only
Takata et al. 2007 [27]	0 of 12	0 of 12	0 of 12	Typical Spitz nevi
Da Forno et al. 2009 [28]	2^h^ of 19	2^i^ of 22	2^9^ of 22	Typical and Atypical Spitz nevi
Emley et al. 2010 [29]	n/a	0 of 20	1 of 20	Typical and Atypical Spitz nevi

Total (%)	24 of 180 (13.3%)	4 of 98 (4.1%)	21 of 330 (6.4%)	

(a) All represent copy number increases of chromosome 11p; (b) all twelve represent copy number increases of chromosome 11p, eight of which contain mutations; (c) scalpel used for microdissection of melanocytes from fixed tissue; (d) did not utilize microdissection techniques to isolate melanocytes; (e) laser capture used for microdissection of melanocytes from fixed tissue; (f) two of the six HRAS mutations occurred in atypical nevi; (g) five of the ten BRAF mutations occurred in atypical inflammatory lesions that likely represented dysplastic nevi; (h) one of the two occurred in an atypical nevus; and (i) one of the two occurred in an atypical nevus with both BRAF and NRAS mutations.

**Table 3:** Acquired nevi.

Study	Nevi included	BRAF	NRAS	NRAS & BRAF
	Total	57 of 70	—	—
	Intradermal	37 of 42		
Pollock et al. 2003 [6]	Compound	16 of 23		
	Dysplastic	4 of 5		

Dong et al. 2003 [33]	Benign melanocytic nevi (unspecified)	17 of 24	0 of 24	0 of 24

Saldanha et al. 2004 [22]	Common acquired nevi	14 of 16	2 of 11	1 of 11

Poynter et al. 2006 [34]	Benign melanocytic nevi (unspecified)	42 of 51	3 of 51	0 of 51

Ichii-Nakato et al. 2006 [10]	Common acquired nevi	105 of 120	—	—

Uribe et al. 2006 [35]	Total	29 of 45	—	—
	Common acquired nevi	16 of 24		
	Atypical nevi	13 of 21		

Bloethner et al. 2007 [36]	Benign melanocytic nevi (unspecified)	18 of 30	—	—

Wu et al. 2007 [12]	Common acquired nevi	83 of 101	—	—

Venesio et al. 2008 [37]	Total	11 of 22	0 of 22	0 of 22
	Compound	6 of 13	0 of 13	0 of 13
	Intradermal	3 of 6	0 of 6	0 of 6
	Junctional	2 of 3	0 of 3	0 of 3

Total		376 of 479 (78.5%)	5 of 108 (4.6%)	1 of 108 (0.9%)

**Table 4:** Blue nevi.

	GNAQ	BRAF	NRAS	HRAS
Yazdi et al. 2003 [7]	—	0 of 20	—	—
Saldanha et al. 2004 [22]	—	3 of 25	0 of 15	—
Van Raamsdonk et al. 2009 [40]	24 of 29	—	—	—

Total	24 of 29 (82.8%)	3 of 45 (6.7%)	0 of 15 (0%)	—

**Table 5:** Summary of common mutations found in congenital, spitz, acquired, and blue nevi.

Classification	Mutations and Relative Frequency
Congenital nevi	NRAS > BRAF
Spitz nevi	HRAS > BRAF > NRAS
Acquired nevi	BRAF > NRAS
Blue nevi	GNAQ > BRAF

## References

[1] Robinson WA, Lemon M, Elefanty A, Harrison-Smith M, Markham N, Norris D (1998). Human acquired naevi are clonal. *Melanoma Research*.

[2] Hui P, Perkins AS, Glusac EJ (2001). Assessment of clonality in melanocytic nevi. *Journal of Cutaneous Pathology*.

[3] Carr J, Mackie RM (1994). Point mutations in the N-ras oncogene in malignant melanoma and congenital naevi. *British Journal of Dermatology*.

[4] Jafari M, Papp T, Kirchner S (1995). Analysis of ras mutations in human melanocytic lesions: activation of the ras gene seems to be associated with the nodular type of human malignant melanoma. *Journal of Cancer Research and Clinical Oncology*.

[5] Papp T, Pemsel H, Zimmermann R, Bastrop R, Weiss DG, Schiffmann D (1999). Mutational analysis of the N-ras, p53, p16(INK4a), CDK4, and MC1R genes in human congenital melanocytic naevi. *Journal of Medical Genetics*.

[6] Pollock PM, Harper UL, Hansen KS (2003). High frequency of BRAF mutations in nevi. *Nature Genetics*.

[7] Yazdi AS, Palmedo G, Flaig MJ (2003). Mutations of the BRAF gene in benign and malignant melanocytic lesions. *Journal of Investigative Dermatology*.

[8] Papp T, Schipper H, Kumar K, Schiffmann D, Zimmermann R (2005). Mutational analysis of the BRAF gene in human congenital and dysplastic melanocytic naevi. *Melanoma Research*.

[9] De Raeve LE, Claes A, Ruiter DJ, Van Muijen GNP, Roseeuw D, Van Kempen LCLT (2006). Distinct phenotypic changes between the superficial and deep component of giant congenital melanocytic naevi: a rationale for curettage. *British Journal of Dermatology*.

[10] Ichii-Nakato N, Takata M, Takayanagi S (2006). High frequency of BRAF mutation in acquired nevi and small congenital nevi, but low frequency of mutation in medium-sized congenital nevi. *Journal of Investigative Dermatology*.

[11] Bauer J, Curtin JA, Pinkel D, Bastian BC (2007). Congenital melanocytic nevi frequently harbor NRAS mutations but no BRAF mutations. *Journal of Investigative Dermatology*.

[12] Wu J, Rosenbaum E, Begum S, Westra WH (2007). Distribution of BRAF T1799A(V600E) mutations across various types of benign nevi: implications for melanocytic tumorigenesis. *American Journal of Dermatopathology*.

[13] Dessars B, De Raeve LE, Morandini R (2009). Genotypic and gene expression studies in congenital melanocytic nevi: insight into initial steps of melanotumorigenesis. *The Journal of Investigative Dermatology*.

[14] Takata M, Saida T (2006). Genetic alterations in melanocytic tumors. *Journal of Dermatological Science*.

[15] Demunter A, Stas M, Degreef H, De Wolf-Peeters C, Van den Oord JJ (2001). Analysis of N- and K-ras mutations in the distinctive tumor progression phases of melanoma. *Journal of Investigative Dermatology*.

[16] Kumar R, Angelini S, Hemminki K (2003). Activating BRAF and N-Ras mutations in sporadic primary melanomas: an inverse association with allelic loss on chromosome 9. *Oncogene*.

[17] Van Dijk MCRE, Bernsen MR, Ruiter DJ (2005). Analysis of mutations in B-RAF, N-RAS, and H-RAS genes in the differential diagnosis of Spitz nevus and spitzoid melanoma. *American Journal of Surgical Pathology*.

[18] Bastian BC, Wesselmann U, Pinkel D, LeBoit PE (1999). Molecular cytogenetic analysis of Spitz nevi shows clear differences to melanoma. *Journal of Investigative Dermatology*.

[19] Bastian BC, LeBoit PE, Pinkel D (2000). Mutations and copy number increase of HRAS in Spitz nevi with distinctive histopathological features. *American Journal of Pathology*.

[20] Palmedo G, Hantschke M, Rütten A (2004). The T1796A mutation of the BRAF gene is absent in Spitz nevi. *Journal of Cutaneous Pathology*.

[21] Mihic-Probst D, Perren A, Schmid S, Saremaslani P, Komminoth P, Heitz PU (2004). Absence of BRAF gene mutations differentiates Spitz nevi from malignant melanoma. *Anticancer Research*.

[22] Saldanha G, Purnell D, Fletcher A, Potter L, Gillies A, Pringle JH (2004). High BRAF mutation frequency does not characterize all melanocytic tumor types. *International Journal of Cancer*.

[23] Gill M, Renwick N, Silvers DN, Çelebi JT (2004). Lack of BRAF mutations in Spitz nevi. *Journal of Investigative Dermatology*.

[24] Turner DJ, Zirvi MA, Barany F, Elenitsas R, Seykora J (2005). Detection of the BRAF V600E mutation in melanocytic lesions using the ligase detection reaction. *Journal of Cutaneous Pathology*.

[25] Fullen DR, Poynter JN, Lowe L (2006). BRAF and NRAS mutations in spitzoid melanocytic lesions. *Modern Pathology*.

[26] La Porta CAM, Cardano R, Facchetti F (2006). BRAF V599E mutation occurs in Spitz and Reed naevi. *Journal of the European Academy of Dermatology and Venereology*.

[27] Takata M, Lin J, Takayanagi S (2007). Genetic and epigenetic alterations in the differential diagnosis of malignant melanoma and spitzoid lesion. *British Journal of Dermatology*.

[28] Da Forno PD, Pringle JH, Fletcher A (2009). BRAF, NRAS and HRAS mutations in spitzoid tumours and their possible pathogenetic significance. *The British journal of dermatology*.

[29] Emley A, Yang S, Wajapeyee N, Green MR, Mahalingam M (2010). Oncogenic BRAF and the tumor suppressor IGFBP7 in the genesis of atypical spitzoid nevomelanocytic proliferations. *Journal of Cutaneous Pathology*.

[30] Dumaz N, Hayward R, Martin J (2006). In melanoma, RAS mutations are accompanied by switching signaling from BRAF to CRAF and disrupted cyclic AMP signaling. *Cancer Research*.

[31] Wojnowski L, Stancato LF, Larner AC, Rapp UR, Zimmer A (2000). Overlapping and specific functions of Braf and Craf-1 proto-oncogenes during mouse embryogenesis. *Mechanisms of Development*.

[32] Smalley KSM, Xiao M, Villanueva J (2009). CRAF inhibition induces apoptosis in melanoma cells with non-V600E BRAF mutations. *Oncogene*.

[33] Dong J, Phelps RG, Qiao R (2003). BRAF oncogenic mutations correlate with progression rather than initiation of human melanoma. *Cancer Research*.

[34] Poynter JN, Elder JT, Fullen DR (2006). BRAF and NRAS mutations in melanoma and melanocytic nevi. *Melanoma Research*.

[35] Uribe P, Andrade L, Gonzalez S (2006). Lack of association between BRAF mutation and MAPK ERK activation in melanocytic nevi. *Journal of Investigative Dermatology*.

[36] Bloethner S, Snellman E, Bermejo JL (2007). Differential gene expression in melanocytic nevi with the V600E BRAF mutation. *Genes Chromosomes and Cancer*.

[37] Venesio T, Chiorino G, Balsamo A (2008). In melanocytic lesions the fraction of BRAF alleles is associated with sun exposure but unrelated to ERK phosphorylation. *Modern Pathology*.

[38] Hussein MRAE, Wood GS (2002). Molecular aspects of melanocytic dysplastic nevi. *Journal of Molecular Diagnostics*.

[39] Blokx WAM, Van Dijk MCRF, Ruiter DJ (2010). Molecular cytogenetics of cutaneous melanocytic lesions—diagnostic, prognostic and therapeutic aspects. *Histopathology*.

[40] Van Raamsdonk CD, Bezrookove V, Green G (2009). Frequent somatic mutations of GNAQ in uveal melanoma and blue naevi. *Nature*.

[41] Yan J, Roy S, Apolloni A, Lane A, Hancock JF (1998). Ras isoforms vary in their ability to activate Raf-1 and phosphoinositide 3-kinase. *Journal of Biological Chemistry*.

[42] Davies H, Bignell GR, Cox C (2002). Mutations of the BRAF gene in human cancer. *Nature*.

[43] Uribe P, Wistuba II, González S (2003). A frequent event in benign, atypical, and malignant melanocytic lesions of the skin. *American Journal of Dermatopathology*.

[44] Maldonado JL, Fridlyand J, Patel H (2003). Determinants of BRAF mutations in primary melanomas. *Journal of the National Cancer Institute*.

[45] Thomas NE, Edmiston SN, Alexander A (2007). Number of nevi and early-life ambient UV exposure are associated with BRAF-mutant melanoma. *Cancer Epidemiology Biomarkers and Prevention*.

[46] Landi MT, Bauer J, Pfeiffer RM (2006). MC1R germline variants confer risk for BRAF-mutant melanoma. *Science*.

[47] García-Borrón JC, Sánchez-Laorden BL, Jiménez-Cervantes C (2005). Melanocortin-1 receptor structure and functional regulation. *Pigment Cell Research*.

[48] Más JS, Gerritsen I, Hahmann C, Jiménez-Cervantes C, García-Borrón JC (2003). Rate limiting factors in melanocortin 1 receptor signalling through the cAMP pathway. *Pigment Cell Research*.

[49] Lin JY, Fisher DE (2007). Melanocyte biology and skin pigmentation. *Nature*.

[50] Lin J, Takata M, Murata H (2009). Polyclonality of braf mutations in acquired melanocytic nevi. *Journal of the National Cancer Institute*.

[51] Van Raamsdonk CD, Fitch KR, Fuchs H, De Angelis MH, Barsh GS (2004). Effects of G-protein mutations on skin color. *Nature Genetics*.

[52] Onken MD, Worley LA, Long MD (2008). Oncogenic mutations in GNAQ occur early in uveal melanoma. *Investigative Ophthalmology and Visual Science*.

[53] Lamba S, Felicioni L, Buttitta F (2009). Mutational profile of GNAQ in human tumors. *PLoS One*.

[54] Bauer J, Kilic E, Vaarwater J, Bastian BC, Garbe C, De Klein A (2009). Oncogenic GNAQ mutations are not correlated with disease-free survival in uveal melanoma. *British Journal of Cancer*.

[55] Chang F, Steelman LS, Shelton JG (2003). Regulation of cell cycle progression and apoptosis by the Ras/Raf/MEK/ERK pathway (Review). *International journal of oncology*.

[56] Medrano EE, Yang F, Boissy R (1994). Terminal differentiation and senescence in the human melanocyte: repression of tyrosine-phosphorylation of the extracellular signal-regulated kinase 2 selectively defines the two phenotypes. *Molecular Biology of the Cell*.

[57] Wellbrock C, Rana S, Paterson H, Pickersgill H, Brummelkamp T, Marais R (2008). Oncogenic BRAF regulates melanoma proliferation through the lineage specific factor MITF. *PLoS One*.

[58] Voice JK, Klemke RL, Le A, Jackson JH (1999). Four human Ras homologs differ in their abilities to activate Raf-1, induce transformation, and stimulate cell motility. *Journal of Biological Chemistry*.

